# Correlation and predictability of ocular aberrations and the visual outcome after quadrifocal intraocular lens implantation: a retrospective longitudinal study

**DOI:** 10.1186/s12886-019-1195-x

**Published:** 2019-08-19

**Authors:** Chia-Yi Lee, Jing-Yang Huang, Chi-Chin Sun, Shun-Fa Yang, Hung-Chi Chen, Hung-Yu Lin

**Affiliations:** 1 Department of Ophthalmology, Show Chwan Memorial Hospital, Changhua, Taiwan; 2 Department of Optometry, College of Medicine and Life Science, Chung Hwa University of Medical Technology, Tainan, Taiwan; 3 Institute of Medicine, Chung Shan Medical University, Taichung, Taiwan; 4 Department of Optometry, Chung Shan Medical University, Taichung, Taiwan; 5 Department of Optometry, Yuanpei University of Medical Technology , Hsinchu, Taiwan; 6 Department of Exercise and Health Promotion, Chung Chou University of Science and Technology, Changhua, Taiwan; 7 Department of Medical Research, Chung Shan Medical University Hospital, Taichung, Taiwan; 8 Department of Ophthalmology, Chang Gung Memorial Hospital, Linkou, Taiwan; 9 Department of Medicine, Chang Gung University College of Medicine, Taoyuan, Taiwan; 10 Center for Tissue Engineering, Chang Gung Memorial Hospital, Linkou, Taiwan; 11 Department of Ophthalmology, Chang Gung Memorial Hospital, Keelung, Taiwan; 12 Department of Chinese Medicine, Chang Gung University, Taoyuan, Taiwan

**Keywords:** Aberration, PanOptix, Intraocular lens, Quadrifocal, Cataract

## Abstract

**Background:**

To evaluate the correlating and predicting factors of visual outcome after implantation of newly developed diffractive quadrifocal intraocular lens (IOL).

**Methods:**

A retrospective longitudinal study was conducted. Patients who underwent diffractive quadrifocal IOL implantation with a follow-up period longer than six months and records of wavefront aberrometer within one week perioperatively were enrolled. Accordingly, a total of 73 eyes from 73 patients were included. The postoperative distance and near visual acuity, ocular aberrations and postoperative symptoms were collected. The correlation and predictability between ocular aberrations and the postoperative visual outcome were evaluated.

**Results:**

The corrected distance visual acuity (CDVA) one month postoperatively was significantly better than the preoperative status, and insignificant improvement was found six months postoperatively. Preoperative Tracey refraction spherical equivalent (TRSE), angle alpha, and spherical aberration (SA) were significantly correlated with postoperative CDVA and near corrected visual acuity (NCVA). For postoperative ocular aberrations, TRSE, angle alpha, and SA were significantly correlated with CDVA six months postoperatively and NCVA, while the trefoil, internal higher order aberration (HOA) and total HOA were associated with NCVA. Preoperative angle alpha could predict all postoperative visual performances, while postoperative TRSE and angle alpha could predict the CDVA six months postoperatively and NCVA. A large angle alpha is associated with visual disturbance and dissatisfaction.

**Conclusion:**

The angle alpha preoperatively and postoperatively was correlated with the postoperative vision and could predict visual outcome in patients who had diffractive quadrifocal IOL implanted. Furthermore, the majority of ocular aberrations were also associated with certain postoperative vision.

## Introduction

Cataracts are the leading cause of reversible blindness and account for approximately 57% of visual impairment in the Chinese population [1]. Surgery is always advocated to treat patients with prominent cataracts for preserving vision [2]. Phacoemulsification accompanied with monofocal intraocular lens (IOL) implantation has been applied for decades and has yielded acceptable visual performance [3]. Despite the good distance visual acuity, the monofocal design leads to poor near visual acuity in more than 50% of patients [4].

In recent years, the multifocal IOL has been widely applied to patients scheduled for cataract surgery [5–7]. The near and intermediate visions are significantly better compared to those of traditional monofocal IOLs, according to previous experiences that patients who have multifocal IOL implanted do not need to wear spectacles [7–10]. Some patients who have multifocal IOL implanted have suffered from halo and glare, thus decreasing the quality of life [11–13]. In addition, patients with irregular astigmatism are less suitable for multifocal IOL due to poor postoperative visual acuity, which may eventually require IOL exchange to relieve visual symptoms [14].

The diffractive quadrifocal IOL (brand name: PanOptix, Alcon, Texas) has been applied currently with good visual outcome [15, 16], and provides a continuous range of vision that is better than that of the previous multifocal IOL [17]. Similar to the other multifocal IOL, the visual acuity and postoperative halos still vary among individuals [18]. Since the higher order aberrations (HOA) are correlated with some complications of multifocal IOL implantation, such as halo, glare and impaired visual acuity [19, 20], a certain relationship that has rarely been investigated before may exist between the ocular aberrations and visual outcome in diffractive quadrifocal IOL.

Herein, we aimed to evaluate the correlating and predicting ocular aberrations of visual outcome after the newly developed diffractive quadrifocal IOL implantation by assessment with a wavefront aberrometer. Both the far and near visual acuities and the preoperative and postoperative ocular aberrations were enrolled in the analysis.

## Materials and methods

### Subjects

The retrospective longitudinal study was conducted in Show Chwan memorial hospital where medical charts had been reviewed. Patients receiving diffractive quadrifocal IOL implantation from 2017 to 2018 who (1) had a follow-up period longer than six months and (2) received a wavefront aberrometer (brand name: iTrace, Tracey Technologies, Texas) examination within one week preoperatively and postoperatively were enrolled in the current study. The exclusion criteria included the following: (1) best-corrected visual acuity equal to or worse than counting finger; (2) diagnosed with ocular tumor; (3) diagnosed with congenital cataracts or traumatic cataracts; (4) received major ophthalmic surgery (e.g., vitrectomy, trabeculectomy, corneal transplantation) before the cataract surgery and during the follow-up period; (5) received refractive procedure, including laser in situ keratomileusis, photorefractive keratectomy, radial keratectomy and phakic IOL implantation, before the cataract surgery and during the follow-up period; and (6) previous corneal scarring, corneal opacity and corneal neovascularization. Only the right eyes of the patients were included in the current study.

### Surgical procedure

All the cataract surgeries were performed by one ophthalmologist (HY Lin) with a standardized procedure. Briefly, femtosecond laser assistance with a single device (LenSx, Alcon, Texas) was applied in all patients for corneal incision, followed by curvilinear capsulorhexis and lens fragmentation. Then, conjunctival sac swab with beta-iodine and prophylactic moxifloxacin eye drop instillation were performed before cataract surgery. After the ophthalmic viscoelastic device injection and hydrodissection, phacoemulsification and aspiration were performed via a single device (Centurion, Alcon, Texas). The diffractive quadrifocal IOL was then implanted, and the corneal incision wound was closed by the hydroseal technique. Moxifloxacin eye drops were administered again after the surgery, and Tobradex ointment was applied for one week postoperatively.

### Ophthalmic examinations

The demography, prominent postoperative complications and subjective satisfaction at the final visit of each patient were recorded from medical documents. The corrected distance visual acuity (CDVA) by Snellen chart because the logarithmic visual acuity chart is not available at our institution, spherical equivalent (SE) and intraocular pressure (IOP) one week before surgery, one month after surgery and six months after surgery, as well as the near corrected visual acuity (NCVA) by the Jaeger near chart six months after surgery, were collected and analyzed. According to our experience in preceding multifocal IOL implantation other than this diffractive quadrifocal IOL, the visual acuity has minimal possibility to change and the short-term visual complication like transient blurred near vision or halo will improve six months postoperatively, thus we arranged NCVA examination six months after surgery. For the ocular aberrations, the wavefront aberrometer exam was performed by a single technician and pupil diameter of 3.0 mm or larger was confirmed before the examination for all patients to ensure a reliable result. The following parameters were yielded after the examination: Tracey refraction spherical equivalent (TRSE), which modified the spherical and the cylinder power according to the eye position, angle alpha, coma, trefoil, spherical aberration (SA), HOA of cornea, HOA of internal eye, HOA of total eye and dysfunctional lens index (DLI). All the ocular aberrations were enrolled in the analysis, and the patients were excluded if any of the above indexes were absent in either preoperative or postoperative examination, or a pupil diameter of at least 3.0 mm could not be achieved even after multiple mydriatic agent instillation. In addition, patients with IOL decentration, which judged by one ophthalmologist (HY Lin), were selected and presented with the basic data, visual acuity and angle alpha in a specific table.

### Statistical analysis

SPSS 20 (SPSS Inc. Chicago, Illinois) was applied for the statistical analysis in the current study. The visual acuity was presented with LogMAR throughout the whole study, and the descriptive analysis was used for all the ocular parameters discussed in the current study. The repeated measure one-way analysis of variance with post-hoc exam was used for the visual acuity, SE and IOP throughout the study period, while the paired t-test was applied to evaluate the difference of ocular aberrations before and after surgery. For evaluating the correlation between both preoperative as well as postoperative ocular aberrations to the visual acuity after the diffractive quadrifocal IOL implantation, a generalized mixed model was utilized with compound symmetry covariance type. The fixed effects in the generalized mixed model included all the ocular aberrations, while the random effects included the preoperative CDVA, SE and IOP. Then, the estimated value and fixed effect were produced. In the next step, a receiver operating characteristic (ROC) curve was used to assess the predictability of each ocular aberration. The area under curve (AUC) was then produced and the coordinate point, defined as the point with a sensitivity more than 0.7 and a “1-specificity” value less than 0.35, was calculated for ocular aberrations with a significant AUC. In addition, the positive predictability was defined as a distance visual acuity greater than 0.8 and a near visual acuity greater than J2, and the absolute value of each ocular aberration was enrolled in the ROC analysis. The analysis between the postoperative ocular aberrations and the CDVA one month postoperatively was not performed due to the short time period between the two events. A *P* value of 0.05 or less was regarded to indicate a statistically significant difference using two-tails probability at 95% confidence intervals; however, only a *P* value less than 0.025 was defined to indicate a statistically significant difference in repeated measure one-way analysis of variance for the prevention of family-wise error. On the other hand, a *P* value less than 0.001 was depicted as *P* < 0.001. The statistical power reached 0.90 under the 0.05 alpha value and medium effect size using G*power version 3.1.9.2 (Heinrich-Heine-Universität, Düsseldorf, Germany).

## Result

We included a total of 73 eyes from 73 patients with a mean age of 64.48 years old and a male to female ratio of 23 to 50. The mean preoperative CDVA was 0.48 ± 0.33 with SE of − 0.66 ± 6.09 diopter (D) and an IOP of 14.66 ± 2.98 mmHg. The CDVA one month postoperatively was significantly better than the preoperative status (0.09 ± 0.10, *P* < 0.001), and a further improvement was found 6 months postoperatively but without significant difference (0.08 ± 0.14, *P* = 0.36). The one-month postoperative SE was decreased compared to the preoperative value (− 0.48 ± 0.57, *P* = 0.02), while the IOP remained the same (13.19 ± 2.76, *P* = 0.25). Six months postoperatively, similar status of SE (− 0.49 ± 0.32, *P* = 0.68) and IOP (14.35 ± 3.37, *P* = 0.34) were found compared to the values one month after surgery, and the NCVA 6 months postoperatively showed a mean value of 0.34 ± 0.47.

The preoperative value and postoperative value of wavefront aberrometer-derived ocular aberrations were evaluated, and there was a significant alteration regarding TRSE and all types of HOA and DLI after cataract surgery (*P* < 0.05, Table 1). Concerning the correlation between ocular aberrations and visual acuity, the preoperative TRSE, angle alpha, and SA were significantly and negatively correlated with the CDVA one month and six months postoperatively as well as the postoperative NCVA. In addition, the corneal HOA was negatively correlated with the CDVA one month postoperatively, while the trefoil and internal HOA were negatively associated with the NCVA (*P* < 0.05) (Table 2). For the postoperative ocular aberrations, the TRSE, angle alpha, and SA were significantly and negatively correlated with the CDVA six months postoperatively and the postoperative NCVA, while the trefoil, internal HOA and total HOA were also negatively associated with the NCVA (*P* < 0.05) (Table 3).

**Table 1:** Preoperative and postoperative ocular aberrations from aberrometry

Ocular aberrations (mean ± SD)	Preoperative	Postoperative	*P* value
TRSE	−1.25 ± 2.23	−0.62 ± 0.56	0.03^a^
Angle alpha	0.33 ± 0.12	0.32 ± 0.12	0.42
Coma	0.17 ± 0.17	0.06 ± 0.08	< 0.001^a^
Trefoil	0.13 ± 0.10	0.05 ± 0.08	< 0.001^a^
SA	0.05 ± 0.08	0.01 ± 0.02	< 0.001^a^
Cornea HOA	0.15 ± 0.17	0.09 ± 0.09	0.01^a^
Internal HOA	0.25 ± 0.15	0.11 ± 0.07	< 0.001^a^
Total HOA	0.30 ± 0.19	0.11 ± 0.08	< 0.001^a^
DLI	5.75 ± 2.59	9.27 ± 1.44	< 0.001^a^

*SD* standard deviation, *TRSE* tracey refraction spherical equivalent, *HOA* higher order aberration, *SA* spherical aberration, *DLI* dysfunctional lens indexes

^a^ denotes significant difference

**Table 2:** Correlation of preoperative ocular aberrations to postoperative visual performance

Ocular aberrations	Estimate1	Fix effect1	*P*1	Estimate2	Fix effect2	*P*2	Estimate3	Fix effect3	*P*3
TRSE	0.73	14.10	< 0.001^a^	0.54	9.45	< 0.001^a^	0.84	12.97	< 0.001^a^
Angle alpha	0.92	99.23	< 0.001^a^	0.75	25.00	< 0.001^a^	0.73	11.26	0.003^a^
Coma	0.01	0.06	0.81	0.07	0.01	0.86	0.20	1.56	0.10
Trefoil	0.12	1.43	0.15	0.13	1.99	0.24	0.35	2.31	0.008^a^
SA	0.57	3.73	0.008^a^	0.72	9.56	< 0.001^a^	0.52	3.98	0.02^a^
Cornea HOA	0.33	5.90	0.004^a^	0.11	2.18	0.12	0.07	1.64	0.63
Internal HOA	0.10	1.22	0.27	0.15	2.08	0.17	0.58	2.22	0.01^a^
Total HOA	0.06	0.12	0.89	0.04	0.10	0.91	0.15	0.66	0.52
DLI	0.13	1.82	0.08	0.15	2.39	0.06	0.12	0.58	0.57

1 = corrected distance visual acuity one month postoperative, 2 = corrected distance visual acuity six months postoperative, 3 = near corrected visual acuity, *TRSE* tracey refraction spherical equivalent, *HOA* higher order aberration, *SA* spherical aberration, *DLI* dysfunctional lens indexes

^a^ denotes significant correlation between ocular aberrations and visual outcome

**Table 3:** Correlation of postoperative ocular aberrations to postoperative visual performance

Ocular aberrations	Estimate1	Fix effect1	*P*1	Estimate2	Fix effect2	*P*2
TRSE	0.65	71.89	< 0.001^a^	0.50	17.48	< 0.001^a^
Angle alpha	0.55	32.87	< 0.001^a^	0.54	17.86	< 0.001^a^
Coma	0.22	1.64	0.20	0.09	1.44	0.24
Trefoil	0.10	1.43	0.23	0.42	3.76	0.008^a^
SA	0.45	22.02	< 0.001^a^	0.32	4.05	0.01^a^
Cornea HOA	0.17	0.81	0.62	0.05	0.93	0.69
Internal HOA	0.05	1.13	0.81	0.47	5.07	0.001^a^
Total HOA	0.08	1.23	0.36	0.36	3.47	0.006^a^
DLI	0.14	0.85	0.91	0.14	1.35	0.33

1 = corrected distance visual acuity 6 months postoperative, 2 = near corrected visual acuity, *TRSE* tracey refraction spherical equivalent, *HOA* higher order aberration, *SA* spherical aberration, *DLI* dysfunctional lens indexes

^a^ denotes significant correlation between ocular aberrations and visual outcome

Regarding predictability, a smaller preoperative angle alpha could positively predict a better outcome for CDVA one month and six months postoperatively, as well as the postoperative NCVA, while smaller preoperative SA could positively predict a better outcome for CDVA one month postoperatively and the postoperative NCVA. In addition, a smaller preoperative coma could positively predict a better outcome for CDVA one month postoperatively (Table 4). For the postoperative status, both a smaller TRSE and angle alpha could positively predict a better outcome for CDVA six months postoperatively and the postoperative NCVA, while the smaller trefoil could positively predict a better outcome for the postoperative NCVA (Table 5).

**Table 4:** Predictability of preoperative ocular aberrations to postoperative visual performance

Ocular aberrations	AUC1	*P*1	Coordinate point1	AUC2	*P*2	Coordinate point2	AUC3	*P*3	Coordinate point3
TRSE	0.455	0.514	NA	0.499	0.991	NA	0.489	0.885	NA
Angle alpha	0.715	0.002^a^	0.327	0.733	0.001^a^	0.349	0.651	0.042^a^	0.349
Coma	0.652	0.027^a^	0.115	0.628	0.077	NA	0.540	0.593	NA
Trefoil	0.630	0.059	NA	0.547	0.518	NA	0.527	0.718	NA
SA	0.679	0.009^a^	0.021	0.591	0.211	NA	0.645	0.049^a^	0.026
Cornea HOA	0.422	0.255	NA	0.497	0.967	NA	0.555	0.456	NA
Internal HOA	0.491	0.893	NA	0.520	0.785	NA	0.586	0.248	NA
Total HOA	0.485	0.832	NA	0.520	0.787	NA	0.596	0.242	NA
DLI	0.488	0.858	NA	0.475	0.729	NA	0.418	0.269	NA

1 = corrected distance visual acuity one month postoperative, 2 = corrected distance visual acuity six months postoperative, 3 = near corrected visual acuity, *AUC* area under curve, *TRSE* tracey refraction spherical equivalent, *HOA* higher order aberration, *SA* spherical aberration, *DLI* dysfunctional lens indexes, *NA* not applicable

^a^ denotes significant predictability between ocular aberrations and visual outcome

**Table 5:** Predictability of postoperative ocular aberrations to postoperative visual performance

Ocular aberrations	AUC1	*P*1	Coordinate point1	AUC2	*P*2	Coordinate point2
TRSE	0.773	< 0.001^a^	0.623	0.756	0.001^a^	0.653
Angle alpha	0.748	0.001^a^	0.321	0.751	0.001^a^	0.321
Coma	0.537	0.605	NA	0.525	0.732	NA
Trefoil	0.513	0.860	NA	0.807	< 0.001^a^	0.069
SA	0.411	0.220	NA	0.611	0.134	NA
Cornea HOA	0.570	0.336	NA	0.557	0.442	NA
Internal HOA	0.640	0.053	NA	0.601	0.174	NA
Total HOA	0.523	0.756	NA	0.571	0.339	NA
DLI	0.498	0.981	NA	0.517	0.815	NA

1 = corrected distance visual acuity 6 months postoperative, 2 = near corrected visual acuity, *AUC* area under curve, *TRSE* tracey refraction spherical equivalent, *HOA* higher order aberration, *SA* spherical aberration, *DLI* dysfunctional lens indexes, *NA* not applicable

^a^ denotes significant predictability between ocular aberrations and visual outcome

The IOL decentration was recorded in five patients and the detailed data of these patients was presented in Table 6. A worse postoperative distance and near visual acuity was found in these patients compared to that of the whole study population, and the angle alpha was also larger in these patients. On the other hand, the age did not show a prominent difference compared to the whole group.

**Table 6:** Characters and visual acuity in patients with intraocular lens decentration

Patient number	Age	Preoperative CDVA	Postoperative CDVA1	Postoperative CDVA2	NCVA	Preoperative angle alpha	Postoperative angle alpha
Patient 1	61–70	0.40	0.22	0.10	0.40	0.38	0.38
Patient 2	71–80	0.52	0.30	0.22	0.46	0.43	0.42
Patient 3	61–70	0.46	0.30	0.30	0.46	0.40	0.40
Patient 4	51–60	0.40	0.15	0.15	0.30	0.36	0.35
Patient 5	61–70	0.52	0.22	0.15	0.40	0.38	0.37

1 = corrected distance visual acuity one month postoperative, 2 = corrected distance visual acuity six months postoperative, *CDVA* corrected distance visual acuity, *NCVA* near corrected visual acuity

Regarding the subjective visual symptoms one month postoperatively, the most common complication was blurred near-vision, which occurred in 13 patients. Other postoperative complications included blurred vision at distance vision (6 patients), diplopia (1 patient), halo (3 patients), glare (4 patients) and sensations of dryness (6 patients). The complications 6 months postoperatively occurred in 7 patients which involved blurred vision at distance vision (2 patients), blurred near-vision (5 patient), halo (3 patients), glare (3 patients) and sensations of dryness (5 patients), which showed a decrement. About the satisfaction, 59 patients (80.8%) felt fully satisfaction while another 12 patients (16.4%) showed partial satisfaction. There were two patients (2.7%) who partially unsatisfied about the visual outcome and no patient (0.0%) stated fully unsatisfied for the postoperative vision.

## Discussion

In our study, the postoperative CDVA was significantly improved compared to the preoperative status in both one-month and six-month after surgery. In previous studies, the mean CDVA was less than 0.1 in LogMAR postoperatively, and the NCVA yielded a similar value [16, 21, 22]. The patients who received diffractive quadrifocal IOL implantation in the current study had a mean CDVA of 0.08 and NCVA of 0.34 six months postoperatively, which are comparable to previous results on the CDVA [16, 21, 22]. The relatively worse NCVA may result from the use of a different tool for the near vision evaluation. The best performance of near vision from the Jaeger near chart was only equal to 0.63 of the decimal value, while a study by Kohnen and García-Pérez used the Early Treatment Diabetic Retinopathy Study chart and obtained an optimal near visual performance of 1.0 of the decimal value [16, 21].

The relationship between ocular aberrations and visual performance after diffractive quadrifocal IOL implantation has rarely been evaluated. The current study demonstrated that the preoperative as well as postoperative TRSE and angle alpha yielded significant estimation value for both far and near vision postoperatively with significant differences. Since the angle alpha is the difference between the visual axis and the center of limbus, a larger angle alpha may lead to poor centration of multifocal IOL, and the decentration of IOL can impair the postoperative visual performance as well as elevate the HOA and SE after cataract surgery [23–25]. Moreover, the TRSE was also related to a worse visual outcome, which further supported the necessity that IOL exchange may be needed to decrease the general SE and improve the related poor visual acuity in previous experience [26, 27]. The DLI was associated with the nuclear opalescence score and indicates the quality of the lens [28, 29], and the insignificant relation may be due to the smooth surgery processes in all the patients. Regarding the HOA, only SA showed a universal correlation to both the postoperative far and near visual acuities, which may because of the SA-related halo and glare [30, 31]. In addition, the residual SA was associated with a worse visual outcome in other types of IOL [32], which could yield a similar correlation in the diffractive quadrifocal IOL. The corneal HOA was correlated with the CDVA, while the trefoil and internal HOA were associated with the NCVA. However, the etiology remains to be elucidated. Interestingly, the total HOA was only correlated with the NCVA, which may be due to the different effects of HOAs that lead to non-significant results.

Except for the correlation, the predictability of different indexes cannot be overlooked, as several biometry indexes play an important role in the IOL power calculation [33, 34]. Regarding the ROC curve analysis in the current study, a preoperative and postoperative angle alpha value less than 0.321 could positively predict a distance visual acuity greater than 0.8 or a near vision acuity greater than J2, which was similar to the direction of the generalized mixed model. The above findings suggest that angle alpha influences the postoperative visual outcome most effectively and universally. The conflicting predictability between preoperative (non-significant) and postoperative (significant) TRSE further demonstrated that residual refractive error is a factor that leads to postoperative visual dissatisfaction in patients who have received multifocal IOL implantation [35]. The fair predictability of trefoil and SA on the postoperative visual acuity also shares a similar trend with the generalized mixed model, while the discordant outcomes regarding coma, corneal HOA and internal HOA in the two different analyses may result from the choice of coordinate points, thus influencing the predictability of each index. Although the postoperative ocular aberrations cannot be modified except laser refractive procedure or IOL exchange is performed, the predictability of such parameters for long-term visual acuity could be used to explain the trend of visual recovery to patients. In clinical practice, it is an important issue to avoid excessive expect from patient and following argument.

For the subjective aspect, impaired near vision, starburst, hazy vision, halo, glare and double vision were reported to exist in some patients who received diffractive quadrifocal IOL implantation [15, 16, 18, 36]. The impaired near vision was the most common postoperative symptom in the current study, which may because the working distance (reading or typing) of human is approximately 25 cm while the nearest focal point of the diffractive quadrifocal IOL is 42 cm [7]. Nonetheless, only two patients that complained of halo, glare and both distance and near vision impairment concurrently felt the complications prominently influenced the quality of life after six months follow up. About the subjective satisfaction, more than 90% of patients reported at least partial satisfaction which indicates an acceptable visual and refractive outcome in the current study. In addition, the mean postoperative CDVA (0.30 and 0.26 sequentially) and NCVA (0.46) in the two patients with partial dissatisfaction were worse than the mean value in the whole population. On the other hand, a worse visual outcome and larger angle alpha were found in patients with IOL decentration compared to those of the gross study group, while the preoperative visual acuity and age in those with IOL decentration were similar to the whole study population which illustrates the severe influence of IOL decentration in multifocal IOL implantation. Interestingly, the two patients with the largest angle alpha (patient 2 and patient 3 in Table 6) were exactly the two that experienced worse postoperative visual acuity, multiple postoperative visual disturbances and felt partially unsatisfied for the postoperative vision. Thus, patients with a large angle alpha are not suggested to have the diffractive quadrifocal IOL implantation to avoid dissatisfaction.

There are some limitations in the current study. First, the retrospective nature leads to the absence of preoperative NCVA. Second, there was no control group, and so whether the correlation between ocular aberrations and visual acuity is specific to the diffractive quadrifocal IOL or is universal for all IOLs cannot be evaluated. Moreover, some optic parameters were not recorded, including contrast sensitivity, defocus curve and modulation transfer function, which might have influenced the statistical analysis.

## Conclusion

In conclusion, the preoperative and postoperative TRSE, angle alpha and SA were negatively correlated with the postoperative visual acuity, while a smaller angle alpha could positively predict better far and near visual outcomes in patient who received diffractive quadrifocal IOL implantation. Also, a large angle alpha may relate to IOL decentration, worse postoperative visual acuity, more visual disturbance and poor patient satisfaction. Furthermore, the majority of ocular aberrations were also associated with postoperative vision in some aspects. Further large-scale studies are needed to verify the distinct contraindications of different types of multifocal IOL based on the value of ocular aberrations.

## Data Availability

The datasets for the analysis of the current study are readily available from the corresponding author on reasonable request.

## Abbreviations

AUC: Area under curve
CDVA: Corrected distance visual acuity
D: Diopter
DLI: Dysfunctional lens index
HOA: Higher order aberration
IOL: Intraocular lens
IOP: Intraocular pressure
NCVA: Near corrected visual acuity
ROC: Receiver operating characteristic
SA: Spherical aberration
SE: Spherical equivalent
TRSE: Tracey refraction spherical equivalent
